# Response to Letter to Editor – Comments on: Sympathetic vasomotor outflow during low‐intensity leg cycling in healthy older males

**DOI:** 10.1113/EP091030

**Published:** 2023-01-12

**Authors:** Keisho Katayama, Shigehiko Ogoh

**Affiliations:** ^1^ Research Center of Health Physical Fitness and Sports Graduate School of Medicine Nagoya University Nagoya Japan; ^2^ Department of Biomedical Engineering Toyo University Kawagoe Japan

1

We wish to express our appreciation for Drs Notarius and Floras's insightful comments concerning our recent published study on sympathetic vasomotor outflow during low‐intensity leg cycling in older males (72 ± 3 years of age, mean ± SD; Katayama et al., 2022). We would like to take this opportunity to respond regarding our work, because our experimental procedure was based, in part, on their previous study (Notarius et al., 2019).

One of the major comments concerns the possible mechanism for the decreased muscle sympathetic nerve activity (MSNA) during leg cycling in our study. Drs Notarius and Floras have suggested that the decrease in MSNA during leg cycling in older males is mediated, in part, by the arterial baroreflex, owing to a greater increase in arterial blood pressure (ABP), secondary to attenuated conduit artery compliance. There is no doubt that the arterial baroreflex contributes to the regulation of sympathetic vasomotor outflow during exercise, and thus, we agree, in part, with their concerns. However, it is well established that the arterial baroreflex resets during dynamic exercise, indicating that the operating point of the arterial baroreflex (resting blood pressure) becomes the exercise‐induced high value of ABP (Raven et al., 2006). Moreover, the moving operating point of ABP in the arterial baroreflex occurs very quickly (Barbosa et al., 2016; DiCarlo & Bishop, 1992; Fisher et al., 2007), and thus the arterial baroreflex does not respond to the exercise‐induced increase in ABP (Raven et al., 2006). Considering this background, the effect of arterial baroreflex control of sympathetic vasomotor outflow during exercise might be minor. Although it has been reported that the cardiopulmonary baroreflex also resets during dynamic exercise (Ogoh et al., 2006), we have shown that the cardiopulmonary baroreflex plays an important role in regulating MSNA outflow at the onset of exercise (Ogoh et al., 2022). Thus, the resetting of the cardiopulmonary baroreflex might not be as rapid as the arterial baroreflex.

In our previous study (Katayama et al., 2014), we measured MSNA in young males performing leg cycling at different pedalling frequencies to alter the muscle pump and central blood volume. This design allowed us to examine whether cardiopulmonary loading modulates sympathetic vasomotor outflow during cycling in young males. Consequently, a significant decrease in MSNA was observed during higher pedalling frequencies (80 r.p.m.) but not lower frequencies (60 r.p.m.), indicating that enhancing the muscle pump and loading cardiopulmonary baroreceptors could inhibit sympathetic vasomotor outflow during leg cycling in young males. This experimental approach might be more appropriate to test whether cardiopulmonary baroreflex control of sympathetic vasomotor outflow during exercise is impaired in older individuals. However, in our recent study, we could not isolate the cardiopulmonary baroreflex control of sympathetic vasomotor outflow because we did not examine MSNA responses at the onset of exercise. Thus, we reanalysed the data in our recent study (Katayama et al., 2022) to identify cardiopulmonary baroreflex control of sympathetic vasomotor outflow during the onset of exercise. The changes in MSNA, ABP and estimated central venous pressure (eCVP) were averaged every 30 s in older males (we averaged the data every 60 s in our published paper, Katayama et al., 2022). At the onset of exercise (0–30 s), MSNA burst frequency (BF) and burst incidence (BI) showed large decreases (Figure 1a,b), while the estimated central venous pressure (eCVP) increased (Figure 1c) and mean arterial blood pressure (MAP) increased (Figure 1d). Although MAP continued to increase from 30 to 60 s, there were no further significant changes in MSNA BF and BI, nor were there any further changes in eCVP. These results demonstrated that the effect of changes in ABP on arterial baroreflex control of sympathetic vasomotor outflow is minimal at the onset of exercise. Thus, in the present study, we believe that the decrease in MSNA was mainly attributable to loading of the cardiopulmonary baroreflex via an increase in venous return despite the change in ABP. Our consideration is supported by several previous studies that reported no difference in MSNA responses during loading and unloading of the cardiopulmonary baroreceptors between younger and older males, in which resting ABP was higher in older males than in younger males (Davy et al., 1998; Tanaka et al., 1999).

**FIGURE 1:**
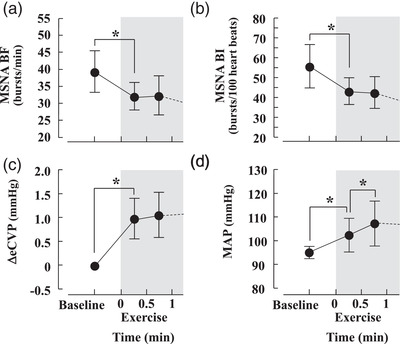
The eCVP responses during exercise are reported as the changes from baseline values. Changes in muscle sympathetic nerve activity burst frequency (MSNA BF; a), muscle sympathetic nerve activity burst interval (MSNA BI; b), estimated central venous pressure (ΔeCVP; c) and mean arterial blood pressure (MAP; d) during cycling in older males. The Bonferroni test was used to evaluate the changes in the variables. ^*^ *P* < 0.05. Values are the mean ± SD

In the study by Notarius et al. (2019), middle‐aged subjects (57 ± 2 years of age, mean ± SEM) exhibited larger decreases in MSNA BI during 0 W one‐legged cycling compared with young subjects (23 ± 1 years of age, mean ± SEM) (−14 ± 2 vs. −9 ± 2 bursts/100 heart beats, mean ± SEM), while there was no difference in the decrease in MSNA BF between the middle‐aged and young subjects (decreased by ∼2–7 bursts/min in each group). In contrast, in our study we observed no difference in the decrease in BI and BF during low‐intensity cycling between younger males (YM; 20 ± 1 years of age, mean ± SD) and older males (OM; 72 ± 3 years of age, mean ± SD) (MSNA BI: YM, −19.9 ± 4.0 vs. OM, −16.1 ± 6.3 bursts/100 heartbeats; and MSNA BF: YM, −10.6 ± 3.3 vs. OM, −8.1 ± 3.8 bursts/min, mean ± SD; Katayama et al., 2022). We considered several between‐study factors that could have contributed to these divergent findings.

Regarding workload, they used 0 W and 30–40% of work rate at peak oxygen uptake (halved for one‐leg) in young and middle‐aged individuals (Notarius et al., 2019). As they have suggested, lower workloads are better suited for examining potential age differences in cardiopulmonary baroreflex control of MSNA. This is because the muscle pump‐induced inhibition of MSNA during cycling is attenuated by muscle metaboreflex activation in an intensity‐dependent manner (Katayama et al., 2020). As such, we also used a low workload (10% of heart rate reserve) in our study, but it appears that the magnitude of the decrease in MSNA with cycling was larger in our study than in the study by Notarius et al. (2019). We speculate that two‐legged cycling in our study (Katayama et al., 2022) induces a larger venous return (i.e., increase in eCVP) than one‐legged cycling (Notarius et al., 2019), resulting in greater loading of cardiopulmonary baroreceptors during two‐legged cycling. This might be the reason for the inconsistent MSNA data.

Regarding sex differences, our study did not include female subjects, which we acknowledge is a major limitation (see Section 4.1 Technical considerations and limitation in the paper by Katayama et al., 2022). The study by Notarius et al. (2019) included 12 males and six females in young‐ and middle‐aged groups, although sex‐specific comparisons were not performed. It is possible that sex differences in cardiopulmonary baroreflex control exist in older populations (Convertino, 1998). However, to our knowledge, there are no data available on whether cardiopulmonary loading‐induced inhibition of MSNA (such as during head‐down tilting or lower‐body positive pressure) is altered by ageing in females. We suggest that further research is needed to clarify this issue of whether sex and ageing affect the cardiopulmonary baroreflex differentially and to examine whether responses to exercise differ between younger and older females.

Other factors related to the sample characteristics might have led to inconsistencies between our studies. First, our subjects (Katayama et al., 2022) were older than the subjects in the study by Notarius et al. (2019) (mean age 72 vs. 57 years, respectively). Additionally, subjects in our study were sedentary, whereas subjects in their study appeared to be recreationally active subjects, hence different fitness levels might be related to the inconsistent results.

The cardiopulmonary baroreflex appears to play a clinically significant role in abnormal cardiovascular responses to exercise. Drs Notarius and Floras and colleagues (Notarius et al., 2015) reported that MSNA increased, rather than decreasing, during mild one‐legged cycling in patients with heart failure. The impaired cardiopulmonary baroreflex might be one of the mechanisms underlying the abnormal sympathoexcitatory response to low‐intensity dynamic leg exercise in these patients. This is supported by literature that demonstrated impaired cardiopulmonary baroreflex control of sympathetic outflow in heart failure patients (Mohanty et al., 1989).

The cardiopulmonary baroreflex could play an important modulatory role in maintaining neural cardiovascular responses to low‐intensity dynamic exercise. Unfortunately, cardiopulmonary baroreflex control during dynamic exercise has seldom been studied. To the best of our knowledge, only a few research groups have attempted to record MSNA during leg cycling to date. It is an important challenge to elucidate the interactive neural regulations (e.g., the arterial baroreflex and cardiopulmonary baroreflex) for changes in sympathetic vasomotor outflow during leg cycling in healthy subjects and patients.

## AUTHOR CONTRIBUTIONS

Both authors have read and approved the final version of this manuscript and agree to be accountable for all aspects of the work in ensuring that questions related to the accuracy or integrity of any part of the work are appropriately investigated and resolved. Both persons designated as authors qualify for authorship, and all those who qualify for authorship are listed.

## CONFLICT OF INTEREST

None declared.

## FUNDING

This work was supported by Japan Society for the Promotion of Science KAKENHI, Grant Numbers: 19H03998 and 22H03479.
